# From bench to bedside: Pursuing equity in precision medicine approaches to pancreatic cancer care

**DOI:** 10.3389/fonc.2022.1086779

**Published:** 2022-12-08

**Authors:** Kelly M. Herremans, Andrea N. Riner, Angel M. Charles, Jeremy A. Balch, Vignesh Vudatha, Devon C. Freudenberger, Ibrahim Nassour, Steven J. Hughes, Jose G. Trevino, Tyler J. Loftus

**Affiliations:** ^1^ Department of Surgery, University of Florida College of Medicine, Gainesville, FL, United States; ^2^ Department of Surgery, Virginia Commonwealth University School of Medicine, Richmond, VA, United States

**Keywords:** diversity, Genomics, equality, translational research, clinical trial

## Introduction

Patients diagnosed with pancreatic cancer face an uncertain future, with an estimated 5-year survival of only 11% (1). Despite continued improvement in the care and treatment of patients diagnosed with pancreatic cancer, it is projected to become the second leading cause of cancer-related death within the next decade (2). To combat this deadly disease, clinicians, researchers, support staff, and patients have worked together to improve quality of care, patient satisfaction, and ultimately, patient outcomes (3). Through the evolution of cancer care, focus has shifted toward a multidisciplinary and precision medicine approach (4). As no two patients are alike, neither are their cancers. To pursue precision medicine in the care and treatment of patients with pancreatic cancer, patient engagement and translational research is critical.

**Figure 1:**
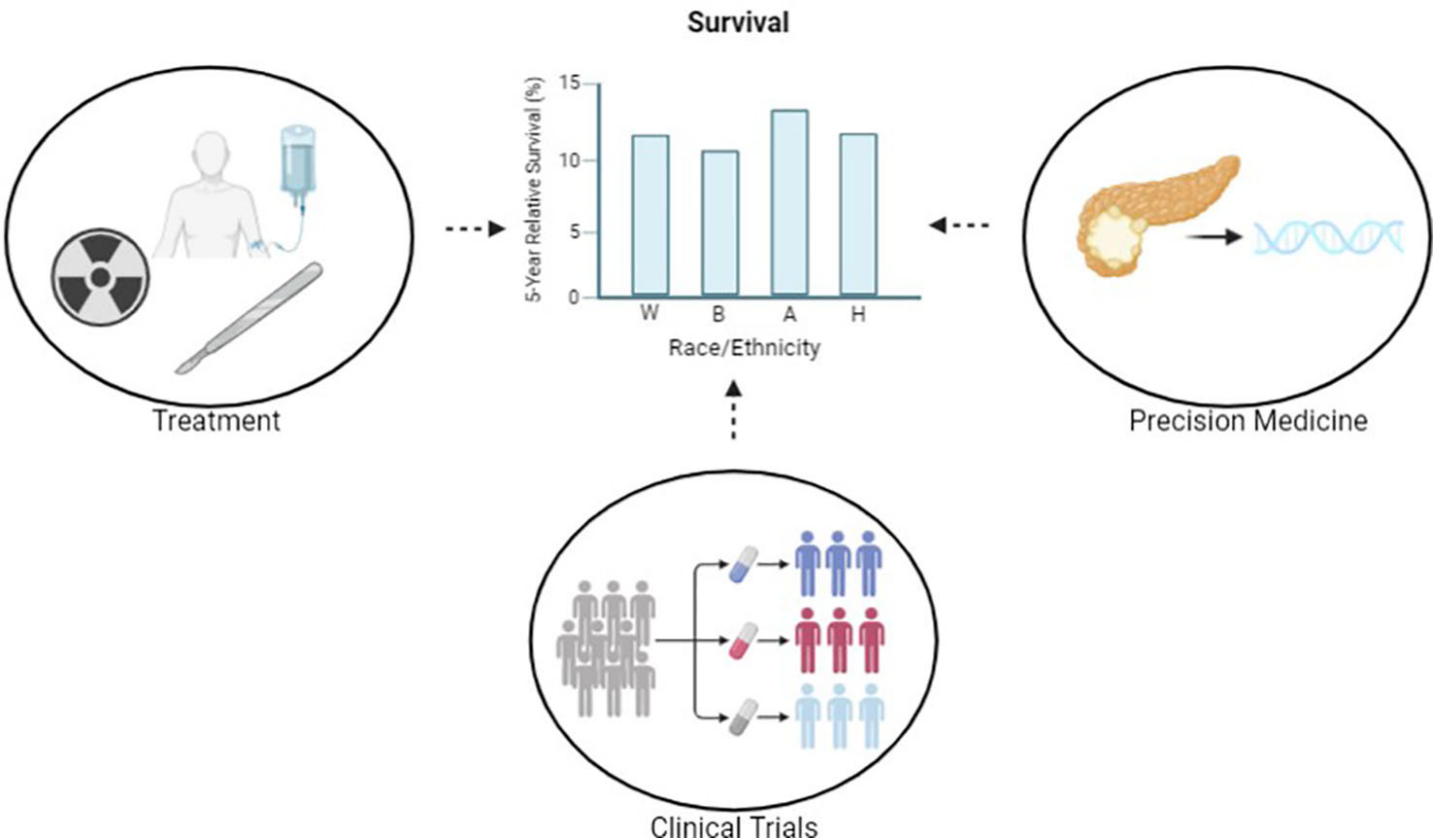
Through the pursuit of equitable care in precision medicine, clinical trials and treatment, we aim to reduce disparities in outcomes. Reported survival statistics are based on SEER 5-year relative survival rates 2012-2018. Created with BioRender.com.

As precision medicine gains traction in pancreatic cancer, it is of the utmost importance to consider how and to whom this approach is applied. Profound health care disparities exist in the diagnosis and treatment of pancreatic cancer. Compared with other races, Black Americans have a higher age-adjusted incidence of pancreatic cancer as well as a higher age-adjusted mortality (**Figure 1**) (1). Furthermore, Black patients are often diagnosed with pancreatic cancer at younger ages and with more advanced disease. Conversely, Asian and Hispanic patients have lower age-adjusted incidence and mortality (1, 5). Health care disparities exist along multiple points of a patient’s pancreatic cancer treatment trajectory, including both diagnosis and treatment (6).

As precision medicine is implemented in the treatment of pancreatic cancer, it is important to address these inequities through the promotion of patient inclusion and provider diversity. In order to promote inclusion in translational research and subsequently precision medicine, genomic datasets as well as clinical trials must include a diverse patient population. Furthermore, to reduce disparities in referrals, provider and systemic bias must be mitigated. In this commentary, we discuss the existing disparities in pancreatic cancer care and how they may be addressed to better the care and treatment of all patients.

## Improving diversity in precision medicine

Improvements in the cost and speed of next-generation sequencing have allowed for the expansion of multiomic datasets in pancreatic cancer research (7). As a result, these data have facilitated the implementation of precision medicine. Precision medicine is thought to become one of the biggest revolutions in the healthcare industry in the near future. It aims to treat patients based on a combination of factors including their genetic make-up, lifestyle and environment and rejects the notion of “one-size-fits all” medicine (8). Through the identification of unique features of tumor and patient biology, targeted therapeutics may be designed specifically for the patient. The application of precision medicine in pancreatic cancer has aimed to best treat the patient while minimizing adverse effects. To achieve this goal, therapeutics are designed based on existing data. Large national –omic datasets are often used to explore the possibilities of targeted therapy. For example, The Cancer Genome Atlas (TCGA) is a federally-funded repository that has become the reference for variant frequencies in human tumors (9). In 2017 alone, TCGA was cited in over 1,000 manuscripts (10). However, within this pancreatic cancer dataset of 185 total patients, only 7 patients (3.8%) were Black, 5 patients (2.7%) were Hispanic or Latinx, and 11 patients (5.9%) were Asian (11).

Additionally, within the cBioPortal for Cancer Genomics, an analysis tool for exploring large-scale genomic datasets, data on race and ethnicity is sparse (12, 13). Within the five individual datasets (ICGC, QCMG, TCGA, UTSW, CPTAC) for pancreatic adenocarcinoma, 70.6% of cases are missing data on race and 48.8% of cases are missing data on ethnicity. However, among those with these data provided, 90.8% of patients were White (11–18).

To provide equitable care, therapeutic discovery must be based on representative sample types. The application of precision medicine in a diverse patient population should be based on inclusive research. Efforts to promote this diversity and inclusion have recently been implemented including broad national efforts such as the All of Us research program and more focused multi-institutional collaborations such as the Florida Pancreas Collaborative (19, 20). These programs promote patient engagement and inclusion to build diverse health databases with the goals of identifying and reducing health care disparities.

## Diversity in pancreatic cancer clinical trials

Diversity in pancreatic cancer clinical trials has remained unchanged over the last two decades. Following the requirement of race and ethnicity reporting in cancer clinical trials, the proportion of pancreatic cancer clinical trials providing these data has improved significantly. However, despite this improvement in data reporting, non-white patients remain consistently underrepresented in pancreatic cancer clinical trials. This lack of patient diversity in cancer clinical trials was identified two decades ago and since that time, efforts toward improving inclusion have had little success (21).

Diversity in pancreatic cancer clinical trials is critical to the application of precision medicine. If cancer therapeutics are not trialed in patients of all backgrounds, they have the potential to disproportionately cause adverse effects in underrepresented minorities (22). This in turn potentiates pancreatic cancer health disparities. Given the complexity of this problem, a multi-faceted approach to promoting diversity must be implemented. To ensure that clinical trials are inclusive of patients from all backgrounds, changes must not only be made by providers and patients but also system-wide.

Currently, there are a number of system-wide barriers that block equitable care in pancreatic cancer clinical trials. A recent study by Riner et al. found that eligibility criteria disproportionally exclude Black patients from pancreatic cancer clinical trials (23). Furthermore, when these criteria are decreased within reason, pancreatic cancer clinical trials become more inclusive (22).

Location of cancer clinical trials also plays a role in the diversity of its trial enrollees. It is important for pancreatic cancer clinical trials to be conducted in areas of diverse representation in order to reduce the barrier of transportation to trial locations. Moreover, community engagement is a central component to improving trial diversity. Studies have shown that minority patients are just as likely as White patients to enroll in a clinical trial as long as they are given the opportunity (21, 24).

To improve referral patterns, bias must be addressed. The integration of bias training has been suggested to lead to modest improvements in referral patterns (25, 26). However, a more sustainable solution to bias is to build a more diverse cancer healthcare workforce. Given only 3% of oncologists are Black and only 4.7% are Hispanic, focused efforts are much needed to improve the diversity of cancer healthcare providers (27).

There is much work to be done to improve the diversity of patients enrolled in pancreatic cancer clinical trials. It is important to continue to pursue these efforts as pancreatic cancer therapy transitions to precision medicine. By creating a more inclusive clinical trial process and subsequently more diverse trial participants, we may reduce healthcare disparities in pancreatic cancer care.

## Promoting inclusion in pancreatic cancer care

Beyond improving diversity in preclinical research and clinical trials, it is even more paramount that patients receive equitable pancreatic cancer care. Though there may be racial and ethnic differences in tumor biology, there are also significant disparities in referral patterns, treatment, and subsequently, outcomes in pancreatic cancer care. Compared with White patients, Black patients diagnosed with pancreatic cancer are less likely to be referred to a medical oncologist (52.6% vs. 60.2%, p<0.001), radiation oncologist (25.6% vs. 32.5%, p<0.05), or a surgeon (72.1% vs. 78%, p ≤ 0.01) (28). Though the reasons for these decreased referral rates remain largely unidentified, some have suggested concern for systemic and provider bias as well as decreased willingness for patients to undergo treatment. To address these disparities comprehensively, the cause for these discrepancies should be identified. Once identified, active effort must be taken to negate these causes.

Once patients are referred to pancreatic cancer specialists, care is not always delivered equitably. In patients with resectable disease, multiple studies have shown that White patients are more likely to be offered and actually undergo surgical resection (28–32). Furthermore, Non-White patients are more likely to receive care from low-volume surgeons, which are often associated with higher rates of morbidity and mortality (33). Hospital-level variation in surgical volume, hospital protocols and provider experience and decision-making ultimately may affect the care a patient receives (6). Following surgical resection, Black patients are more likely to have higher rates of morbidity and are less likely to be referred for adjuvant chemotherapy (29, 34). In the approximately 80% of patients that are found to have advanced disease at diagnosis, notable racial and ethnic disparities exist. Black and Hispanic patients are less likely than White patients to receive chemotherapy and radiation for pancreatic cancer (35). Unfortunately, likely as a result of these treatment discrepancies, Black patients have repeatedly been found to have worse overall survival compared with White patients diagnosed with pancreatic cancer (1).

## Discussion

As the care and treatment of patients with pancreatic cancer transitions into precision medicine, it is crucial that it is delivered equitably. In the future, care of patients with pancreatic cancer will be tailored to their unique disease characteristics. Multidisciplinary tumor boards have been increasingly utilized to gain insight from different perspectives (36). Patient cases are discussed among healthcare providers from different specialties to provide patients with the best treatment plan for their specific disease. Furthermore, dedicated pancreas hospital units have been designed to incorporate a multidisciplinary approach to pancreatic cancer care (37–39). The development and implementation of these disease specific units have allowed for improved flow of information from “bench to bedside.”

It is important that these multi-disciplinary care teams further the pursuit of equitable care in patients with pancreatic cancer. Starting at “the bench,” diverse representation and inclusion in preclinical studies is critical. To diversify these patient samples, patient engagement and interinstitutional collaboration are key. In the first step toward equitable care, all patients should be approached for recruitment into clinical data repositories in a manner that builds rapport and overcomes mistrust. Conscious effort should be placed into mitigating consent bias by appropriately describing the collection process, emphasizing the patient’s role in decision making, discussing the importance of the research project along with the risks and alternatives to the collection process (40). To promote equity and ultimately improve patient outcomes, a trusted relationship between provider and patient must be established. Patient care must be patient-centric, which includes developing plans with the patient instead of for the patient. This in turn also encourages patient engagement in research endeavors (41).

Furthermore, collaboration amongst institutions promotes inclusion of a diverse patient population. Through the involvement of patients in different locations, patients of a variety of backgrounds may be reflected in preclinical studies. Additionally, researchers and clinicians from different locations may contribute their unique experiences. By including a diverse group of patients in the studies that ultimately dictate cancer therapeutics, pancreatic cancer health disparities may be partially mitigated.

Following the design of pancreatic cancer therapeutics, it is also critical to incorporate a diverse population in the trials that test their efficacy. Through the identification and elimination of the practices that instill bias, including the existence of stringent eligibility criteria, a diverse population may be represented in pancreatic cancer clinical trials (23). This becomes paramount as cancer treatment transitions to precision medicine. If therapeutics are not trialed on the populations in which they are prescribed, they have the potential to have increased adverse effects and subsequently perpetuate pancreatic cancer health disparities.

Most importantly, we must strive to reduce pancreatic cancer health disparities in the care and treatment of patients. As multidisciplinary tumor boards and pancreas units are designed to improve the quality of care for patients with pancreatic cancer, it is crucial that they are designed to improve the care of all patients. With a recent shift toward centralization of pancreatic cancer care in pancreas units within high volume hospitals, it is critical that these centers are inclusive of patients from racial and ethnic minority backgrounds. In order to do so, patient, provider, and systemic barriers to equitable care will need to be addressed. The cost of high-quality care, transportation to and from hospitals, and referral patterns should be accessible for all patients. Furthermore, systemic bias must be addressed when centralizing pancreatic cancer care. It is essential to consider healthcare disparities in the realm of pancreatic cancer when these healthcare changes are implemented. Equitable care in pancreatic cancer should always be the standard of care.

## Author contributions

KH drafted and edited this manuscript. AR, AC, JB, VV, DF, IN, SH, JT, TL participated in reviewing and editing. TL provided guidance, oversight and funding for this manuscript. All authors contributed to the article and approved the submitted version.
